# Changes in lipid metabolism convey acid tolerance in *Saccharomyces cerevisiae*

**DOI:** 10.1186/s13068-018-1295-5

**Published:** 2018-10-29

**Authors:** Zhong-peng Guo, Sakda Khoomrung, Jens Nielsen, Lisbeth Olsson

**Affiliations:** 1 Department of Biology and Biological Engineering, Industrial Biotechnology, Chalmers University of Technology, 412 96 Gothenburg, Sweden; 2 Department of Biochemistry and Siriraj Metabolomics and Phenomics Center, Faculty of Medicine Siriraj Hospital, Mahidol University, Bangkok, Thailand; 3 Department of Biology and Biological Engineering, Systems and Synthetic Biology, Chalmers University of Technology, 412 96 Gothenburg, Sweden; 4 Novo Nordisk Foundation Center for Biosustainability, Technical University of Denmark, Building 220, 2800 Kongens Lyngby, Denmark; 5 Present Address: LISBP, INSA, INRA, CNRS, Université de Toulouse, Toulouse, France

**Keywords:** Weak acids, Sustainable, Yeast physiology, *S. cerevisiae*, Oxidative stress

## Abstract

**Background:**

The yeast *Saccharomyces cerevisiae* plays an essential role in the fermentation of lignocellulosic hydrolysates. Weak organic acids in lignocellulosic hydrolysate can hamper the use of this renewable resource for fuel and chemical production. Plasma-membrane remodeling has recently been found to be involved in acquiring tolerance to organic acids, but the mechanisms responsible remain largely unknown. Therefore, it is essential to understand the underlying mechanisms of acid tolerance of *S. cerevisiae* for developing robust industrial strains.

**Results:**

We have performed a comparative analysis of lipids and fatty acids in *S. cerevisiae* grown in the presence of four different weak acids. The general response of the yeast to acid stress was found to be the accumulation of triacylglycerols and the degradation of steryl esters. In addition, a decrease in phosphatidic acid, phosphatidylcholine, phosphatidylserine and phosphatidylethanolamine, and an increase in phosphatidylinositol were observed. Loss of cardiolipin in the mitochondria membrane may be responsible for the dysfunction of mitochondria and the dramatic decrease in the rate of respiration of *S. cerevisiae* under acid stress. Interestingly, the accumulation of ergosterol was found to be a protective mechanism of yeast exposed to organic acids, and the *ERG1* gene in ergosterol biosynthesis played a key in ergosterol-mediated acid tolerance, as perturbing the expression of this gene caused rapid loss of viability. Interestingly, overexpressing *OLE1* resulted in the increased levels of oleic acid (18:1n-9) and an increase in the unsaturation index of fatty acids in the plasma membrane, resulting in higher tolerance to acetic, formic and levulinic acid, while this change was found to be detrimental to cells exposed to lipophilic cinnamic acid.

**Conclusions:**

Comparison of lipid profiles revealed different remodeling of lipids, FAs and the unsaturation index of the FAs in the cell membrane in response of *S. cerevisiae* to acetic, formic, levulinic and cinnamic acid, depending on the properties of the acid. In future work, it will be necessary to combine lipidome and transcriptome analysis to gain a better understanding of the underlying regulation network and interactions between central carbon metabolism (e.g., glycolysis, TCA cycle) and lipid biosynthesis.

**Electronic supplementary material:**

The online version of this article (10.1186/s13068-018-1295-5) contains supplementary material, which is available to authorized users.

## Introduction

Weak organic acids such as acetic, formic and levulinic acids are present in lignocellulosic hydrolysate as potential inhibitors that can hamper the use of this renewable resource for fuel and chemical production [1]. The yeast *Saccharomyces cerevisiae* plays an essential role in the fermentation of lignocellulosic hydrolysates. However, this yeast species is also a food spoilage agent when it gains resistance against the currently used organic-acid preservatives [2]. Therefore, it is essential to understand the underlying mechanisms of acid tolerance of this yeast either for developing robust industrial strains, or for controlling spoiling yeasts.

The effects of weak acids on *S. cerevisiae* have been generally ascribed to acidification of the cytosol by the protons released and/or accumulation of the anions of the acid, which can be toxic to essential metabolic functions [3, 4]. Acetic acid in particular inhibits NADH dehydrogenase and induces programmed cell death [5, 6]. Lipophilic weak acids, such as sorbate and benzoate which are commonly used as preservatives in the food and beverage industry, can damage the membrane and disrupt oxidative phosphorylation [7, 8], influence the transportation of nutrients [9], and trigger the endogenous production of superoxide free radicals [10]. Responses to weak acids, such as ATP-dependent efflux of the protons and anions, via plasma membrane H^+^-ATPase Pma1p and the ATP-binding cassette transporter (Pdr12p), have been suggested [11, 12]. The involvement of H^+^-ATPase and Pdr12p at the expense of ATP compromises biomass formation [13].

To develop more robust biocatalysts with high acetic-acid tolerance, metabolic engineering [14–17], genome shuffling [18], evolutionary engineering [19] and genome-wide gene screening [20, 21] have been used. Despite these efforts, there is still a need to develop strains of *S. cerevisiae* tolerant to acetic acid and/or other acids. Plasma-membrane remodeling has recently been suggested to play a role in the acetic-acid adaptation of *S. cerevisiae* and *Zygosaccharomyces bailii* [22–26]. Particularly, sphingolipids have been shown to play an important role in acetic-acid resistance in *Z. bailii* [24, 25]. However, the mechanism responsible and the physiological significance of cell-membrane remodeling in response to acid stress remain largely unexplored.

The main components of the cell membrane of *S. cerevisiae* are glycerophospholipids, sterols and intra-membrane proteins [27, 28]. In addition, yeast cells have a pool of neutral lipids consisting of triacylglycerols (TAGs) and steryl esters (STEs), stored as lipid droplets that serve as reservoirs of cellular energy and building blocks for membrane lipids. The most abundant fatty acid (FA) species of the yeast cells are oleic acid (C18:1n-9) and palmitoleic acid (C16:1n-7), followed by palmitic acid (C16:0) and stearic acid (18:0), and small amounts of myristic acid (C14:0) and arachidic acid (C20:0) [27]. Quantitative studies of the response of neutral lipids and cellular FAs under conditions of acid stress may help to increase our knowledge on lipid metabolism under specific growth conditions. In the present study, we have analyzed both lipids and fatty acids in *S. cerevisiae* exposed to stress from different acids, i.e., hydrophilic acetic, formic, levulinic acid and lipophilic cinnamic acid. The aim of this study was to map the changes in the lipid profile of the yeast cells when exposed to weak acids with different properties, and to guide the genetic engineering of yeast to control its robustness in acid stress.

## Results

### Physiological response of *S. cerevisiae* to weak acids

Under the reference condition (without addition of acid), yeast started to grow on glucose without a lag phase, at *μ*_max_ reaching 0.41 h^−1^ followed by a second growth phase on the ethanol produced during the glucose growth phase (Table 1). Yeast growth stopped immediately following addition of the acids. It was noted that 0.17 mM undissociated cinnamic acid, a much smaller amount than the other acids, led to a 50% reduction in the biomass yield (Table 1), which indicates that the hydrophobicity of the acid governs the toxicity of the acid. Less undissociated formic acid (10.0 mM) was required to give the same level of biomass reduction as 68.7 mM acetic acid and 79.0 mM levulinic acid. Formic acid has a lower hydrophobicity than acetic and levulinic acids, but its higher toxicity has been ascribed to its smaller molecular size [29, 30].

**Table 1:** Effects of weak acids on the growth of *S. cerevisiae* under aerobic conditions

	Control	Acetic acid	Cinnamic acid	Formic acid	Levulinic acid
pKa	N/A	4.79	4.44	3.75	4.66
Log*P*^a^	N/A	− 0.17	2.13	− 0.54	− 0.49
Concentration (mM)	0	180	0.7	180	260
Undissociated acid (mM)	0	68.7	0.15	10.0	79.0
Adaptation phase Glc. (h)	0	32	4	24	48
*μ*_max-glc_ (h^−1^)	0.41 ± 0.01	0.18 ± 0.01	0.12 ± 0.01	0.20 ± 0.01	0.22 ± 0.01
Yx/*s* (g-DCW/g-glc)	0.15 ± 0.01	0.07 ± 0.01	0.07 ± 0.01	0.07 ± 0.00	0.07 ± 0.01
Adaptation phase EtOH. (h)	0	60	N/A	32	54

*N/A* not available, *Glc* glucose, *EtOH* ethanol

^a^Log*P*, the lipophilic tendency given by the partition coefficient octanol–water (P)

The growth of yeast on glucose and ethanol in the presence of the organic acids was greatly impaired, as can be seen from the long lag phases and low growth rates (Table 1, Additional file 1: Fig. S1). In addition, it was noted that glucose and ethanol were continuously consumed by acid-stressed cells during the adaptation phase on either of the carbon sources, and that this was not accompanied by any accumulation of biomass. No obvious decrease in acid concentration was observed in any of the cultures during the adaptation phase on glucose (Additional file 1: Fig. S1). Strikingly, growth was not resumed for the yeast exposed to cinnamic acid. Moreover, yeast cells exposed to acid stress exhibited a significant decrease in specific rates of O_2_ consumption, compared to the control (Table 2). In addition, increases were observed in the specific rates of glucose consumption, and ethanol and CO_2_ production in response to formic, acetic and levulinic acids stress. However, these specific rates were lower in cells exposed to cinnamic acid than the control, reflecting the acid-dependent inhibition of glycolysis and respiration. After the adaptation on ethanol, yeast started to grow on acetic acid only after ethanol depletion. It is unclear why the presence of ethanol represses the consumption of acetic acid. As for formic acid-stressed cells, formic acid was co-consumed with ethanol. In *S. cerevisiae*, formic-acid consumption is catalyzed by NAD^+^-dependent formate dehydrogenases, which oxidize formate to carbon dioxide and H_2_O, without energy generation [31]. In this case, the biomass was mainly produced from ethanol. By contrast, yeast was unable to consume levulinic acid (Additional file 1: Fig. S1).

**Table 2:** Metabolic flux analysis of *S. cerevisiae* in the presence of weak acids at pH 5 under aerobic conditions

	Control	Acetic acid	Cinnamic acid	Formic acid	Levulinic acid
O_2_ (mmol/g/h)	11.6 ± 0.2	7.3 ± 0.2	6.0 ± 0.0	8.3 ± 0.2	7.8 ± 0.2
Glucose (mmol/g/h)	18.1 ± 0.2	18.6 ± 0.2	15.9 ± 0.1	18.9 ± 0.6	19.4 ± 0.3
CO_2_ (mmol/g/h)	29.0 ± 0.4	29.4 ± 0.1	20.0 ± 0.2	29.3 ± 0.5	30.3 ± 0.4
Ethanol (mmol/g/h)	26.5 ± 0.2	28.8 ± 0.2	17.9 ± 0.3	29.6 ± 0.4	30.1 ± 0.3

### Comparison of neutral lipid storage

**Fig. 1:**
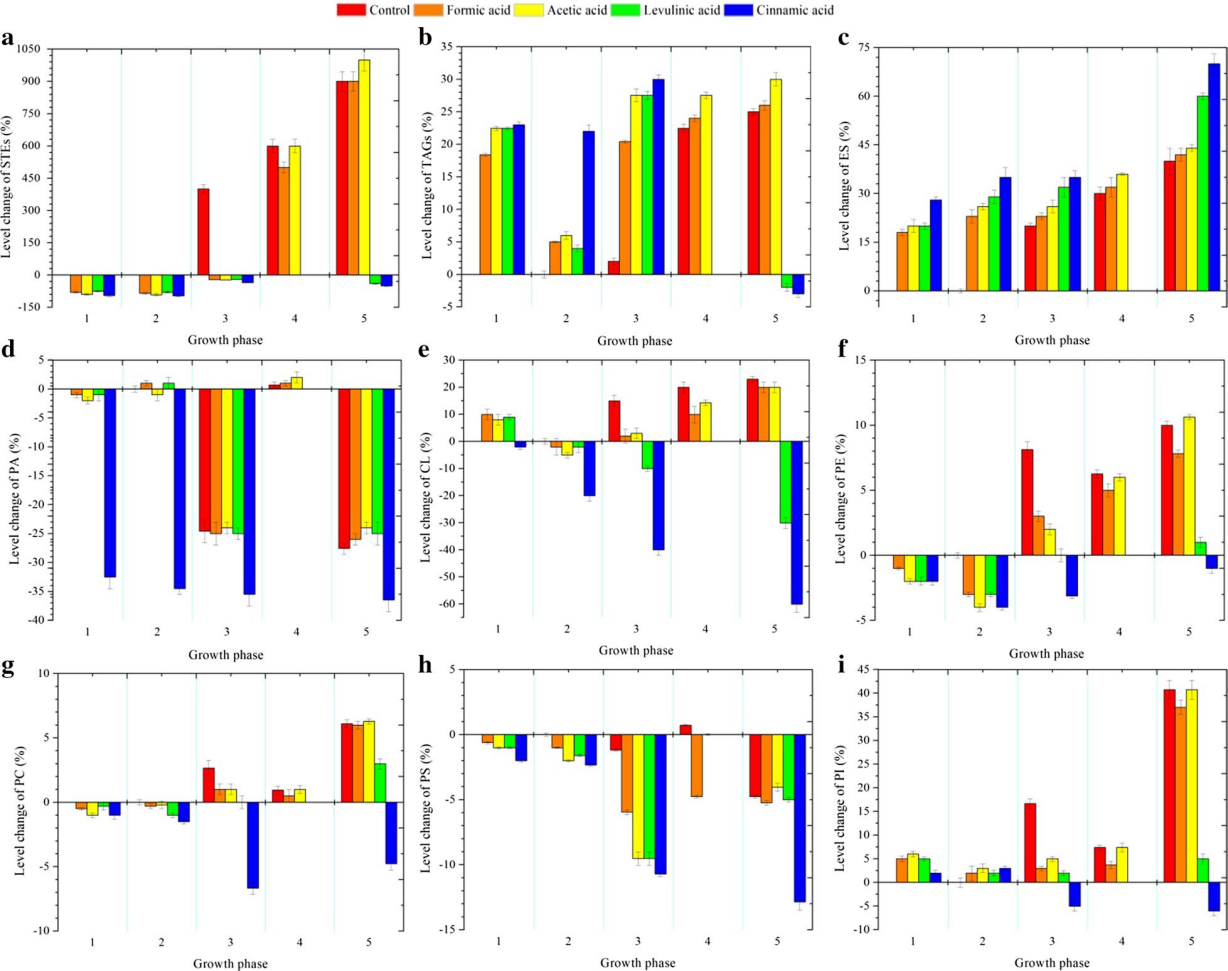
Comparison of the lipidome profiles of the yeast strains during aerobic culture without and with the addition of acetic, formic, levulinic and cinnamic acids, at five different growth phases, at pH 5.0. **a** STEs, **b** TAGs, **c** ES, **d** PA, **e** CL, **f** PE, **g** PC, **h** PS and **i** PI. The growth phases are defined as: phase 0, the exponential growth phase before acid addition; phase 1, the adaptation phase on glucose after acid addition; phase 2, the exponential growth phase on glucose; phase 3, the adaptation phase on ethanol; phase 4, the exponential growth phase on ethanol; and Phase 5, the stationary phase. Level change = (lipid content of phase 1–5—lipid content of phase 0)/lipid content of phase 0

The amount of neutral lipid storage in lipid droplets is generally relatively low in *S. cerevisiae* (< 15%), but is probably highly dynamic as yeast is readily and rapidly able to adjust its internal metabolism according to the growth conditions [32]. The cellular content of STEs decreased by 18% in cells exposed to formic, levulinic and acetic acids, and by 25% in cells under the stress of cinnamic acid during the adaptation phase on glucose (phase 1). Thereafter, a continuous decrease in STEs was observed in the cells grown on glucose (phase 2) and during adaptation on ethanol (phase 3), especially the cells exposed to levulinic and cinnamic acids, for which the decrease in STEs was up to 40% and 50%, respectively, in the stationary phase (phase 5). However, the control adapted on ethanol (phase 3) showed about a 20% increase in cellular STE content compared with cells in the exponential phase (Fig. 1a).

In contrast, the cellular TAG content increased from 18 to 23% in cells under acid stress compared with the control in the adaption phases on glucose and ethanol. During the growth phase on glucose, TAGs were rapidly mobilized in yeast cells exposed to formic, acetic and levulinic acids, but not cinnamic acid. However, a 30% increase in TAG content was observed for the cells exposed to acetic acid in the stationary phase. In comparison, in the stationary phase, with the depletion of the carbon sources in media, the presence of levulinic and cinnamic acids imposed a continuous requirement for ATP generation. As one way to supply energy, degradation of FAs from TAGs to β-oxidation led to a further decrease in the cellular TAG content of those cells [33] (Fig. 1b).

### Comparison of the cellular ergosterol content

The ergosterol content in the cell membrane of *S. cerevisiae* changed considerably under acid stress. While the control showed a gradual decrease in ergosterol from the exponential phase to the stationary phase, exposure of the cells to acids led to continuous accumulation of the sterol content during different growth phases. Specifically, yeast cells exposed to cinnamic acid showed a continuous increase in cellular ergosterol content, ranging from 28 to 70% throughout the cultivation process, followed by cells subjected to levulinic-acid stress, for which an increase from 20 to 60% was observed. Similarly, an increase in cellular ergosterol content was observed in cells exposed to formic acid and acetic acid before glucose depletion. However, none of these three acids resulted in a significant increase in ergosterol in cells grown on ethanol, and the cells contained similar contents of ergosterol to the control during the stationary phase (Fig. 1c).

### Mapping of the cellular phospholipid profile

Exposure of the yeast cells to formic, levulinic and acetic acids did not lead to a significant change in the cellular content of phosphatidic acid (PA) compared to the control at the different growth phases. Remarkably, more than a 30% decrease in cellular PA content was observed in yeast cells exposed to cinnamic acid throughout cultivation (Fig. 1d).

Yeast cells exposed to acetic, formic and levulinic acids showed a 10% increase in the cellular content of cardiolipin, and after acid adaptation, the growing cells contained slightly less cellular cardiolipin than the control. However, a continuous decrease in cardiolipin content, up to 60%, was observed in cells stressed by cinnamic acid throughout cultivation. Less decrease (up to 30%) was observed in the cells exposed to levulinic acid, and only after depletion of the glucose or ethanol (Fig. 1e). The cellular contents of phosphatidylethanolamine (PE), phosphatidylcholine (PC) and phosphatidylserine (PS) decreased in yeast cells under acid stress during the two adaptation phases, and the exponential growth phase on glucose (phase 2), compared to the control (Fig. 1f–h). However, the cellular content of phosphatidylinositol (PI) in yeast cells subjected to acid stress increased during the adaptation phase on glucose (phase 1) and the two growth phases (phase 2 and 4), and decreased during the adaption phase on ethanol (phase 3), compared to the control (Fig. 1i).

### FAs and the unsaturation index of FAs in response to weak acids

**Fig. 2:**
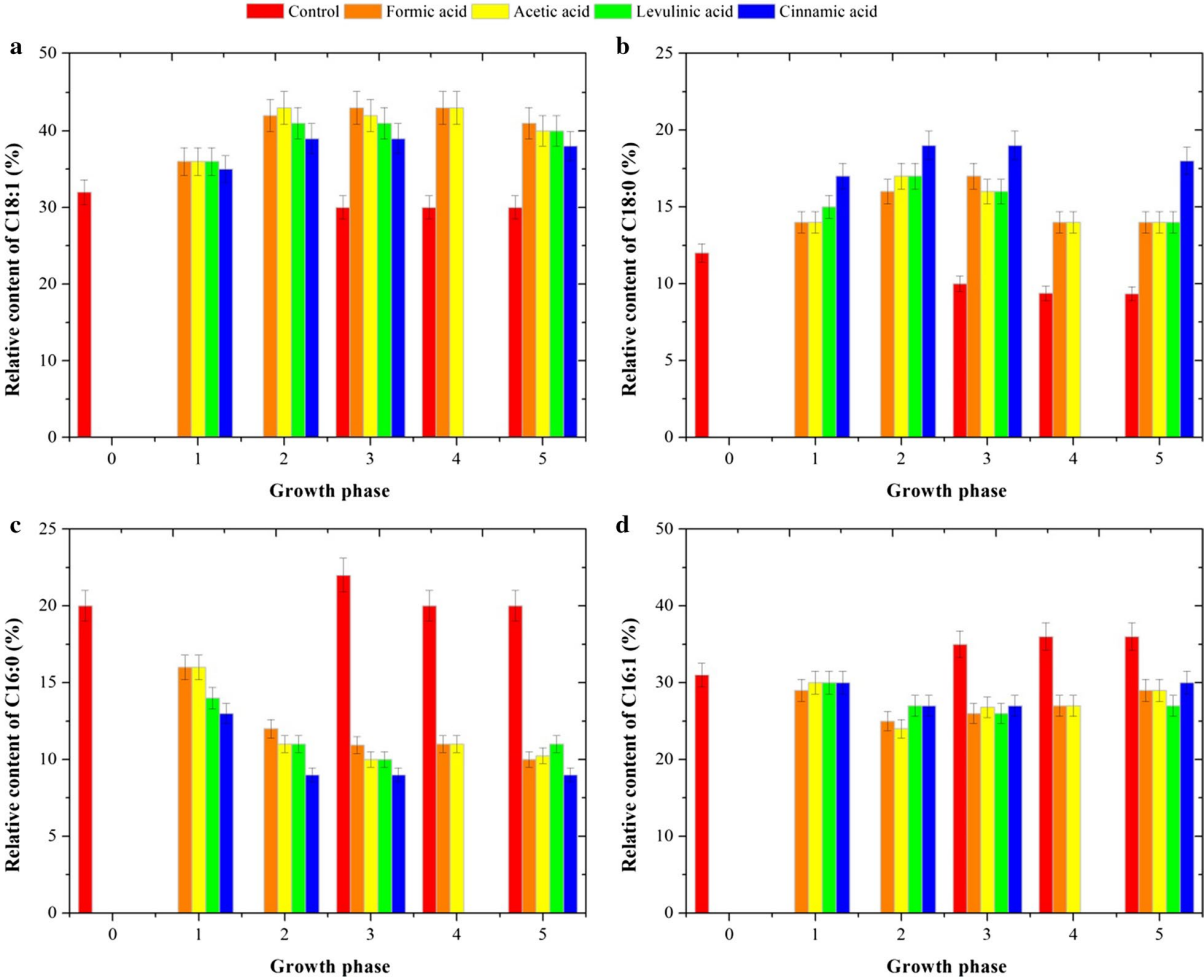
Comparison of the profiles of the abundant fatty acids in polar lipids of yeast strains during aerobic culture without and with the addition of acetic, formic, levulinic and cinnamic acids, at six different growth phases, at pH 5.0. **a** Oleic acid, **b** stearic acid, **c** palmitic acid and **d** palmitoleic acid

Interestingly, in contrast to the relatively small change in the cellular content of phospholipids, exposure of the yeast cells to different acids triggered a significant rearrangement of FA composition of the phospholipids. Concerning the profile of the FAs obtained from polar lipids, mainly phospholipids, in yeast cells under acid stress, the amount of C14:0 was around 5% of the total FAs, similar to that of the control. However, a decrease in C16:0 and C16:1n-7, and an increase in C18:1n-9 and C18:0 were observed for all the acid-stressed cells throughout cultivation. It was noticed in particular that yeast cells exposed to cinnamic acid showed a smaller increase in C18:1n-9 (up to 30%) and a smaller decrease in C16:0 (up to 13%), but a greater increase in C18:0 (up to 90%) and a greater decrease in C16:1n-7 (up to 55%), than the cells stressed by the other acids (Fig. 2). Moreover, about 2% lignoceric acid (C24:0) was found in yeast cells exposed to cinnamic acid, while this FA was negligible in the cells exposed to the other acids and in the control.

Although the FA composition showed significant differences, comparing the content of unsaturated FAs illustrated that the unsaturation index was largely unaffected in the cells exposed to cinnamic acid, compared with the control. However, exposure of the cells to the other acids resulted in an continuous increase in the unsaturation index of FAs compared with the control throughout cultivation (Table 3).

**Table 3:** Effects of weak acids on the unsaturation index of fatty acids under aerobic conditions

	Control	Acetic acid	Cinnamic acid	Formic acid	Levulinic acid
Initial growth phase	62.5 ± 0.8	62.5 ± 0.8	62.5 ± 0.8	62.5 ± 0.8	62.5 ± 0.8
Adaptation glucose	N/A	66.0 ± 1.0	64.0 ± 1.2	65.6 ± 1.0	66.5 ± 0.9
Growth phase on glucose	63.5 ± 1.0	67.0 ± 0.6	65.2 ± 0.6	67.0 ± 0.3	66.8 ± 1.0
Adaptation ethanol	66.0 ± 0.7	69.1 ± 0.0	65.3 ± 1.0	69.0 ± 0.6	67.0 ± 0.3
Growth phase on ethanol	66.0 ± 1.0	70.0 ± 0.4	N/A	70.0 ± 1.1	N/A
Stationary phase	67.0 ± 1.6	69.0 ± 0.8	66.0 ± 2.0	70.0 ± 0.6	68.0 ± 1.0

### Overexpression and repression of *OLE1* in *S. cerevisiae*

**Fig. 3:**
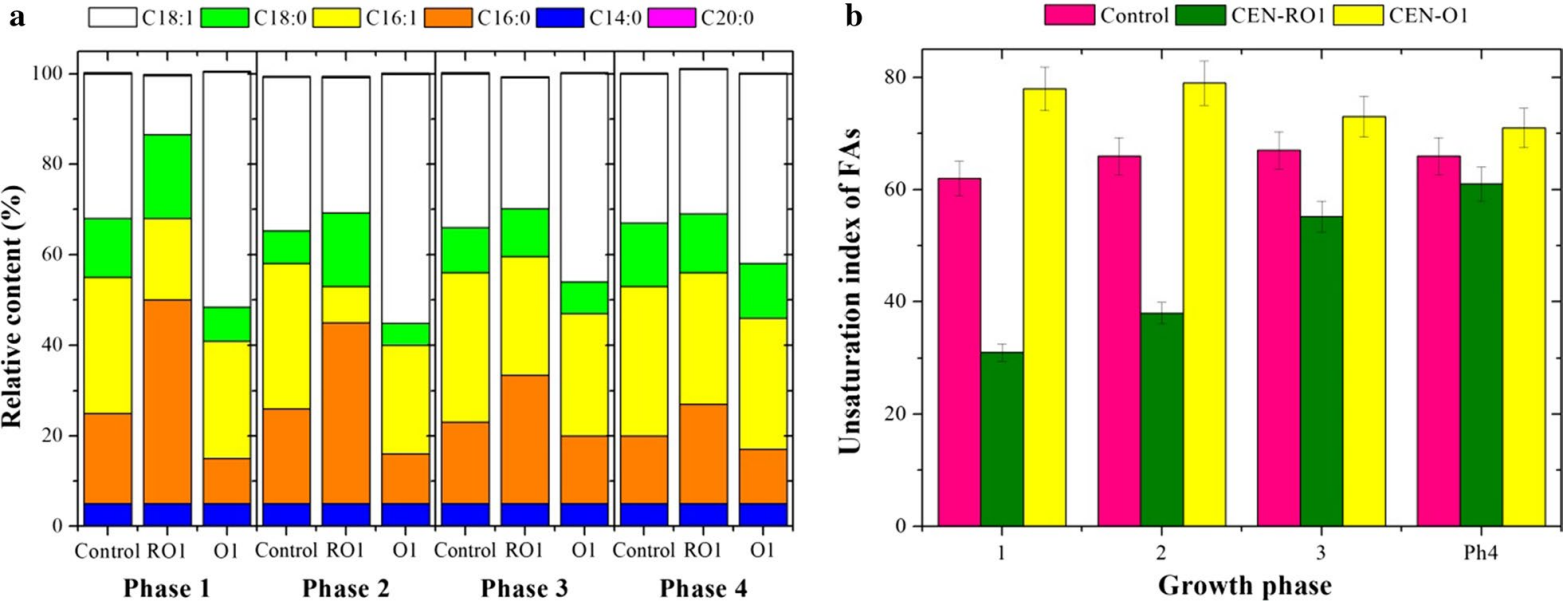
Fatty acid profile (**a**) and unsaturation index (**b**) of the control and recombinant *S. cerevisiae* strains CEN-RO1 (*P*_*TEF*_-*OLE1*-reverse) and CEN-O1 (*P*_*TEF*_-*OLE1*) during the exponential growth phase on glucose (phase 1), lag phase on ethanol (phase 2), ethanol growth phase (phase 3) and stationary phase (phase 4). The unsaturation index was calculated as sum of weight of FA multiplied by the number of unsaturated bonds for each FA in the mixture

The *OLE1* gene encodes the only ∆-9 fatty acid desaturase in *S. cerevisiae* and it is required for the production of monounsaturated FAs [34]. To investigate whether the increase in the unsaturation index of FAs is a protective mechanism of yeast cells in response to acid stress, FA desaturase was overexpressed or repressed in *S. cerevisiae* CEN.PK 113-5D. Under normal growth conditions, the recombinant *S. cerevisiae* CEN-RO1 (*P*_*TEF*_-*OLE1*-reverse) in which *OLE1* is repressed, and *S. cerevisiae* CEN-O1 (*P*_*TEF*_-*OLE1*) in which *OLE1* is overexpressed, showed similar growth patterns to the control strain CEN.PK 113-5D harboring plasmid p426TEF (data not shown). In addition, comparing the FA composition of the phospholipids showed that the amount of C14:0 was largely unchanged in yeast cells in which *OLE1* was overexpressed or repressed, compared with the control. Interestingly, a significant increase in C18:1n-9 and a considerable decrease in C16:0 were observed in yeast cells overexpressing *OLE1*, while yeast cells in which the expression of *OLE1* had been repressed showed a dramatic increase in C16:0 and a significant decrease in C18:1n-9 (Fig. 3a). The unsaturation index of FAs in yeast cells overexpressing *OLE1* increased by 26%, 20%, 9% and 8% in the exponential phase, ethanol adaption phase, ethanol growth phase and stationary phase, respectively, compared with the control. In contrast, the repression of *OLE1* led to a 50% decrease in the unsaturation index of FAs in yeast cells growing on glucose, compared to the control. However, a smaller decrease in the unsaturation index of FAs was seen in this yeast during the other growth phases (Fig. 3b). Therefore, the increase/decrease in the unsaturation index of cells in which *OLE1* was overexpressed or repressed was mainly due to the increase/decrease in cellular content of C18:1n-9.

### The composition and unsaturation index of FAs and acid tolerance of the yeast

**Fig. 4:**
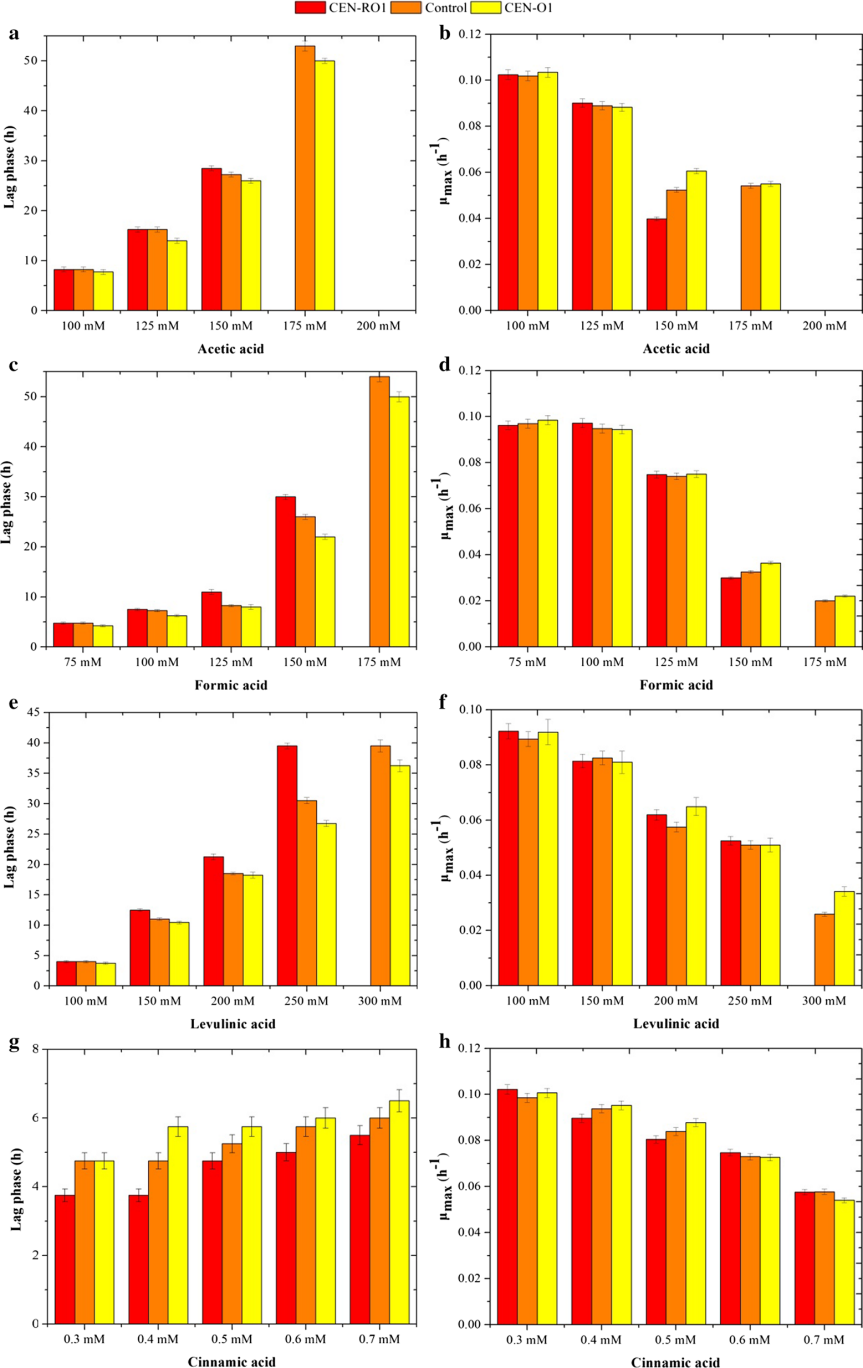
The lag phase and μ_max_ of the control and recombinant *S. cerevisiae* strains CEN-RO1 (*P*_*TEF*_-*OLE1*-reverse) and CEN-O1 (*P*_*TEF*_-*OLE1*) during aerobic growth with the addition of 100–200 mM acetic acid (**a**, **b**), 75–175 mM formic acid (**c**, **d**), 100–300 mM levulinic acid (**e**, **f**) and 0.3–0.7 mM cinnamic acid (**g**, **h**), at pH 5.0

Yeast cells in which *OLE1* is overexpressed or repressed were inoculated into cultures in which formic, acetic, levulinic and cinnamic acids had been added. Increasing the unsaturation index of FAs had a beneficial effect in that it reduced the lag phase and improved the survival rate of the yeast cells exposed to formic, acetic and levulinic acids (Fig. 4, Additional file 1: Fig. S2). However, a significant change in *μ*_max_ was observed, compared to the control. In contrast, yeast cells with a lower unsaturation index of FAs under the stress of formic, acetic and levulinic acids showed a longer lag phase and lower survival rate than the control (Fig. 4, Additional file 1: Fig. S2). When the unsaturation index of FAs was reduced, yeast was unable to grow in the presence of 175 mM formic acid, 175 mM acetic acid and 300 mM levulinic acid. Interestingly, yeast cells in which the unsaturation index of FAs decreased showed a shorter lag phase and higher survival rate than the control under cinnamic acid stress (Fig. 4g, h, Additional file 1: Fig. S2).

### Ergosterol and acid tolerance of the yeast

**Fig. 5:**
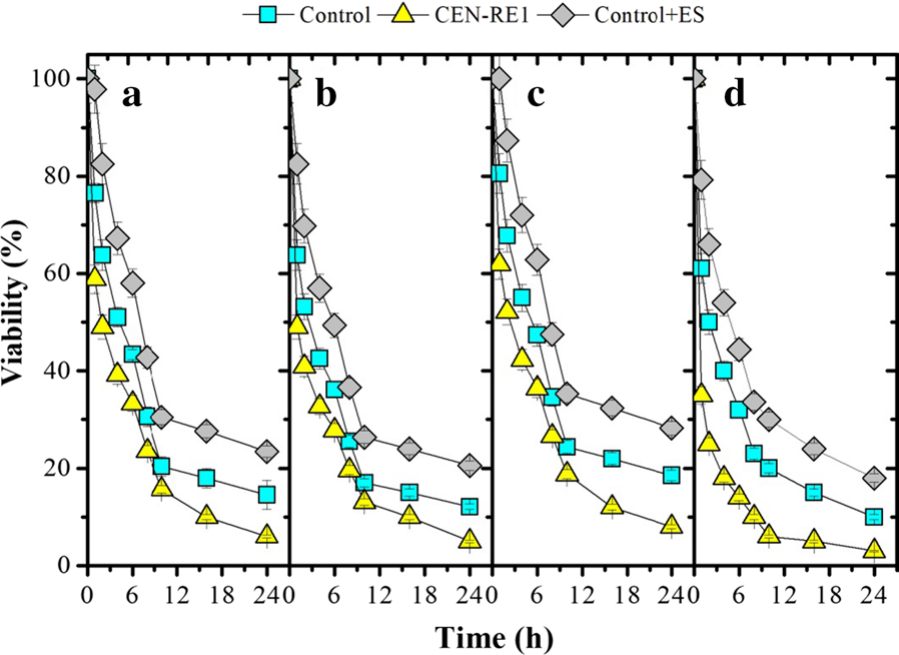
Viable fractions of the *S. cerevisiae* control, the control supplemented with 10.0 μg/ml ergosterol and the recombinant strain CEN-RE1 (*P*_*TEF*_-*ERG1*-reverse) under the stress of: (a) 150 mM acetic acid, (b) 150 mM formic acid, (c) 200 mM levulinic acid and (d) 0.8 mM cinnamic acid, at pH 5.0

To investigate whether the accumulation of ergosterol is a protective mechanism in yeast cells in response to acid stress, the ergosterol biosynthesis pathway was perturbed by repressing the expression of squalene epoxidase (*ERG1*), which plays an essential role in the ergosterol biosynthesis pathway. The cellular content of ergosterol in recombinant *S. cerevisiae* CEN-RE1 (*P*_*TEF*_-*ERG1*-reverse), in which *ERG1* was repressed, decreased by 50% during exponential growth on glucose, compared to the control. In addition, knocking down the expression of this enzyme catalyzing the epoxidation of squalene to 2, 3-oxidosqualene impaired the growth of the yeast on glucose (data not shown). Moreover, *S. cerevisiae* CEN-RE1 was more sensitive to acid stress than the control, as the exposure of *S. cerevisiae* CEN-RE1 to different acids resulted in rapid loss of viability (Fig. 5).

Despite the presence of 10.0 μg/ml ergosterol, the cellular content of ergosterol in cells under the non-stressed condition was largely unaffected. However, the accumulation of ergosterol, up to 10.0 mg/g dry cell weight (DCW), was observed for cells subjected to acid stress, i.e., a 30% increase compared with the control under the stress conditions. In addition, yeast cells with a higher cellular ergosterol content were more resistant to acid stress than the control as they showed a higher survival rate under 24-h acid stress (Fig. 5).

## Discussion

**Fig. 6:**
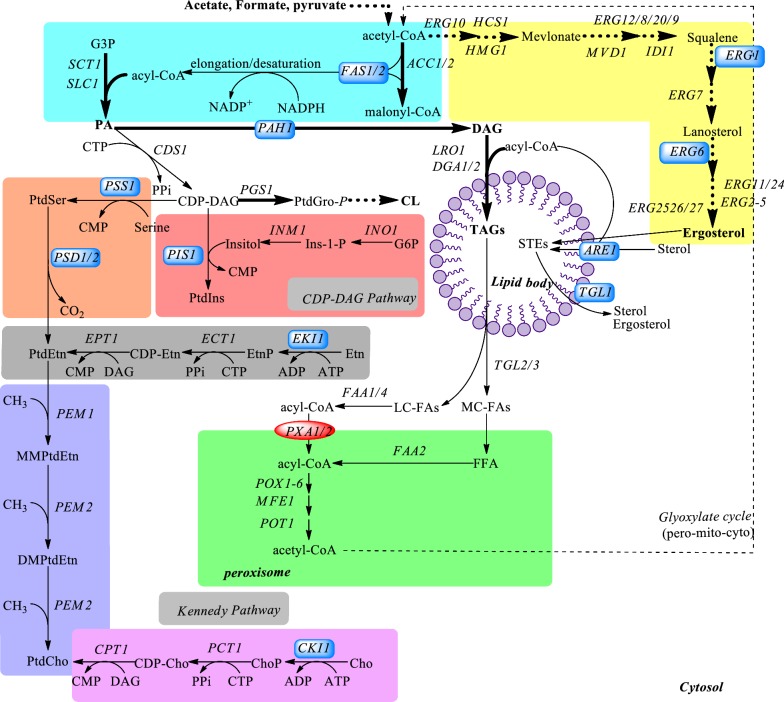
Overview of lipid remodeling in yeast during acid adaptation, where heavy arrows indicate enhanced biosynthesis. Model created from the data available for *S. cerevisiae*. Abbreviations used for major metabolic intermediates are: *G3P* glycerol-3-phosphate, *CDP-DAG* cytidine diphosphate-diacylglycerol, *TAGs* triacylglycerols, *STEs* steryl esters, *PA* phosphatidic acid, *PC* phosphatidylcholine, *CL* cardiolipin, *PE* phosphatidylethanolamine, *PI* phosphatidylinositol, *PS* phosphatidylserine, *ES* ergosterol, *FFA* free fatty acids, *LC-FAs* long-chain fatty acids, *MC-FAs* medium-chain fatty acids. Key gene names refer to the following encoded enzymatic activities: *SCT* glycerol-3-phosphate acyltransferase, *SLC* LPA acyltransferase, *ACC* acetyl-CoA carboxylase, *ARE* acyl-CoA:cholesterol acyltransferase, *DGA* acyl-CoA:DAG acyltransferase, *FAS* fatty acid synthetase, *LRO* phospholipid:diacylglycerol acyltransferase, *MFE* multifunctional enzyme, *PAP* phosphatidate phosphatase, *FAA* fatty acyl-CoA synthetase, *PIS* phosphatidylinositol synthase, *PSS* phosphatidylserine synthase, *PSD* phosphatidylserine decarboxylase, *EKI* ethanolamine kinase, *CKI* choline kinase, *ERG1* squalene epoxidase, *ERG6* squalene reductase, *POT* thiolase, *POX* acyl-CoA oxidase, *PXA* peroxisomal acyl-CoA transporter, *TGL* triacylglycerol lipase. The changes in the expression levels of several key genes (inside the dark blue box) were verified by qPCR. The arrows with dashed lines indicate that multiple reactions are involved in the corresponding synthetic pathway

The lipid remodeling in *S. cerevisiae* during acid adaptation is summarized in Fig. 6. The biosynthesis and hydrolysis of nonpolar lipids (TAGs and STEs) play an important role in cellular FA composition and sterol homeostasis [35]. Indeed, enhanced biosynthesis and the accumulation of neutral lipids have been observed in yeast exposed to environmental stress and starvation [36, 37]. As acid causes dysfunction of the mitochondria and impairs respiration [33], the high specific rate of glucose uptake accompanied by extremely low rate of respiration (almost negligible during acid adaptation as determined by oxygen consumption in this study) causes the accumulation of the intermediates of glycolysis and the TCA cycle. For instance, accumulation of acetyl-CoA, glycerol-3-phosphate and dihydroxyacetone phosphate was observed in acid-stressed cells (data not shown), which may have contributed to the storage of TAGs. Another important contribution to the increase in TAG synthesis lies in the transcriptional regulation of lipid metabolism. For instance, yeast cells exposed to acetic-acid stress showed up-regulation of the *PAH1* gene, which favored the conversion of PA into TAG, and down-regulation of the genes involved in the synthesis of PE, PS and PC (*PSS1*, *PSD1*, *EKI1* and *CKI1*), which indirectly supports TAG accumulation, as their synthesis could compete for the intermittent PA (Fig. 1, Additional file 1: Fig. S3).

Interestingly, yeast cells exposed to weak acids showed down-regulation of FA synthase 1 (Fas1p), which plays a key role in acyl-CoA production (the precursor for PA and TAG biosynthesis) (Additional file 1: Fig. S3). It is unclear how the PA and TAG biosynthesis was favored when the expression of *FAS1* decreased at regulation level. It has been shown that exposure of yeast cells to H_2_O_2_ stress induced a decrease in both Fas expression and activity in the evolved cells. In addition, deletion of one of the FAS alleles, which caused a 50% reduction in Fas activity, led to an increase in the resistance of yeast to H_2_O_2_ [38]. As a follow-up to this observation, the cell-membrane composition was explored to investigate the relation between the reduction of FAS activity and H_2_O_2_ resistance, and the accumulation of very-long-chain fatty acids (VLC-FAs) lignoceric acid (C24:0) (40%) and cerotic acid (C26:0) (50%) was found in the plasma membrane of the mutant cells. The authors, therefore, ascribed the H_2_O_2_ resistance to the fact that a high content of VLC-FAs reduces the overall or localized plasma-membrane permeability to H_2_O_2_ through interdigitation or by modulating the formation of lipid rafts [38]. Yeast cells exposed to weak acids suffered from oxidative stress induced by acids (Additional file 1: Fig. S4); the increase in VLC-FA content of the plasma membrane is probably a defense response of yeast to acid stress [39]. Indeed, VLC-FAs are the precursors of sphingolipid biosynthesis, and the accumulation of VLC-FAs is expected to increase the cellular sphingolipid content and its complexity [26]. Sphingolipids are essential structural components of cellular membranes, in particular the plasma membrane [40]. Recent studies have suggested a link between high levels of complex sphingolipids and the intrinsic tolerance of *Z. bailii* species to acetic acid [23–25]. However, the correlation between the decrease in the expression of Fas and the increase in VLC-FA content needs to be further elucidated.

Using comparative functional genomics analysis, it has been found in a previous study that yeast with a higher tolerance to acetic acid has more oleic acid in the plasma membrane [15]. Our findings confirmed that a higher level of cellular oleic acid contributes to the tolerance of *S. cerevisiae* to acetic, formic and levulinic acids (more hydrophilic), but was detrimental in cells exposed to cinnamic acid (which is more lipophilic). Given the similar property of cinnamic acid to those used as preservatives, such as sorbate and benzoate, in the food and beverage industry, reducing oleic acid content and/or unsaturation index of fatty acids in cell membrane is expected to be useful strategies to impair the survivability of the spoilage yeasts. It remains to be elucidated on the molecular and structural levels, how membrane remodeling influences the FA composition, the degree of saturation and unsaturation. Further work is needed to study how these changes influence the properties of the cell membrane in terms of permeability, integrity and rigidity, either individually or collectively. It is unclear how the change in the expression level of fatty acid desaturase (*OLE1*) was able to significantly influence the cellular content of C18:1n-9 and C16:0.

Cardiolipin is an important phospholipid, known to maintain membrane potential and the architecture of the mitochondria, and provides essential structural and functional support to several proteins involved in mitochondrial bioenergetics [41]. Cardiolipin is particularly susceptible to peroxidation due to the abundance of double bonds in its structure [42], and its close association with respiratory chain proteins, which are known to be a major source of ROS in the mitochondria [43]. Acid stress induces oxidative stress, and lipid peroxidation could cause the loss of cardiolipin content in the mitochondria. Therefore, the enhancement of cardiolipin biosynthesis may partially compensate for the loss of cardiolipin and stabilize the mitochondria in cells stressed by acetic, formic and levulinic acids. However, peroxidation and loss of cardiolipin cannot be avoided in the case of cinnamic acid, due to its ability to cause cell-membrane disruption and oxidative stress. Therefore, preventing cardiolipin loss is probably important in maintaining the normal function of the mitochondria in cells under acid stress.

Free ergosterol is mainly incorporated into the plasma membrane and is responsible for structural properties of the membrane such as fluidity and permeability [44]. Earlier studies have reported a positive correlation between heat sensitivity and ergosterol levels, and that ergosterol contributes to the ethanol tolerance of *S. cerevisiae* [45, 46]. In addition, changes in sterol composition from ergosterol to ergosta5, 8-diene-3-ol have been suggested to contribute to the HCl tolerance of the evolved strains [47]. The *ERG1* gene, encoding squalene epoxidase which catalyzes the epoxidation of squalene to 2, 3-oxidosqualene, has been suggested to be the rate-limiting enzyme in ergosterol biosynthesis [48]. In the present study, we demonstrated for the first time that higher cellular levels of ergosterol improve the viability of yeast cells under acid stress, and repressing the expression level of *ERG1* suggested that the *ERG1* gene played a key role in ergosterol-mediated acid tolerance. The disruptive effect of weak acids on the cell membrane has been known for a long time [4]. The presence of ethanol can exhibit a synergistic inhibitory effect on yeast cells, as a consequence of the effect both the acid and ethanol have on the cell membrane [49]. Therefore, the accumulation of ergosterol may protect the cell membrane against acid stress, as a high level of ergosterol prevents interdigitation and maintains an optimal membrane thickness, as has already been described under ethanol stress [50]. Ergosterol can be produced either by the degradation of STEs, which liberates ergosterol and sterol precursors, or by de novo ergosterol synthesis [51, 52]. Although the sterol intermediates released by the hydrolysis of STEs may be converted into ergosterol much faster than de novo sterol synthesis [53], given the fact that the STE pool is very small when ergosterol is needed for membrane formation during exponential growth, the decrease in the STE pool alone can hardly contribute to the high accumulation of ergosterol in yeast cells under acid stress. In addition, the idea that acid stress enhances de novo ergosterol synthesis is in agreement with our observations that the *ERG1* and *ERG6* genes involved in the ergosterol biosynthetic pathway were up-regulated, and the *TGL1* gene for STE degradation was slightly down-regulated, which further confirmed the important role of *ERG1* in ergosterol-mediated acid tolerance. The *ARE1* gene-encoding sterol esterase was also down-regulated (Additional file 1: Fig. S3). A recent study has revealed that yeast cells under acetic-acid stress contained less ergosterol in the mid-exponential growth phase than non-stressed cells [23]. As yeast physiology is highly dependent on the environmental conditions, the physiological responses obtained in current study may be different from those generated under other growth conditions in the previous study [23]. Differences seen include acid addition from the beginning of culture, cell samples at a different growth phase, and different acid concentration, which determines the toxicity of the acid. Given the complex nature of sterol metabolism, a better understanding of the mechanisms underlying ergosterol biosynthesis is required to design suitable engineering strategies to improve the acid tolerance of yeast.

## Materials and methods

### Yeast strains and media

The haploid, prototrophic *S. cerevisiae* strain CEN.PK 113-7D (MATa) was grown in a defined medium containing vitamins, trace elements and salts including: 7.5 g/l (NH_4_)_2_SO_4_, 3.5 g/l KH_2_PO_4_ and 0.7 g/l Mg_2_SO_4_·7H_2_O with 30 g/l glucose [54]. *S. cerevisiae* CEN.PK 113-5D (*MATa*, *SUC2*, *MAL2*-*8 c*, *ura3*-*52*) was cultured in YPD medium containing 20 g/l peptone, 10 g/l yeast extract and 20 g/l glucose.

### Growth conditions and acid pulse

The yeast was pre-cultured in defined medium (as described above) until the exponential growth phase. Batch cultures were carried out in a 3-l DASGIP bioreactor (DASGIP Biotools LLC, Shrewsbury, MA) with a working volume of 2 l. The temperature was set to 30 °C and the pH was maintained at 5.0 by the automatic addition of 2.0 M KOH. To prevent excessive foaming, 0.30 ml silicone antifoam (Sigma A8311) was added. Aeration was set to 0.5 vvm, and the stirring speed to 600 rpm to give a dissolved oxygen tension of at least 60% of air saturation throughout fermentation. Yeast cells were first cultivated until the optical density at 600 nm (OD) reached 1.0 (exponential growth phase), after which, acetic, formic, levulinic or cinnamic acid was added to the medium. The concentration of each acid added was intended to result in half the biomass yield obtained on glucose under aerobic conditions, as determined by preliminary experiments (Table 1). During the cultivation process, CO_2_ production or O_2_ consumption was measured continuously using an off-gas analyzer.

### Determination of substrate and extracellular metabolites

Cell suspensions (two of 1.5 ml each) were rapidly transferred from the culture into liquid nitrogen. The frozen suspension was thawed on ice. Samples were centrifuged at 3000×*g* for 5 min at 4 °C, and the supernatants were subsequently subjected to high-performance liquid chromatography (HPLC). The measurement conditions used for glucose, glycerol and ethanol, and acetic, formic and levulinic acids were the same as in our previous work [29]. Cinnamic acid was measured using GC–MS, as described previously [55].

### Dry weight determination

Two 10 ml culture samples were filtered through pre-weighed polyethersulfone filters (0.45 μm, Sartorius Biolab, Germany). The biomass retained by the filters was washed, dried in a microwave oven at 150 W for 15 min, and then placed in a desiccator before being weighed.

### Calculation of physiological parameters

All data are presented as the mean ± standard deviation (SD) of biological replicates (*N* ≥ 3). Lag phase was estimated using DMFIT (http://www.ifr.ac.uk/safety/DMfit), as described previously [56]. The biomass yield was obtained as the slope of the linear curve when plotting the biomass concentration versus the glucose concentration during exponential growth on glucose. The specific rates of substrate consumption and product formation were calculated as described previously [57]. The evaporation rate of ethanol was determined in a separate cell-free experiment, and all data were corrected for the evaporation of ethanol (1% of the ethanol at each point).

### Lipid extraction

Yeast cells were harvested at different growth phases and centrifuged at 3000×*g* for 5 min at 4 °C to collect the biomass. The samples were then immediately frozen in liquid nitrogen and placed in a freeze-dryer at − 40 °C overnight before analysis. Lipids were extracted from the yeast cells using a microwave-assisted method as described previously [58]. Briefly, freeze-dried cells (~ 10 mg) were suspended in 7 ml of a mixture of chloroform and methanol (2:1, v/v) containing 50 μg cholesterol as internal standard in a Pyrex borosilicate glass tube (16 × 100 mm). The samples were flushed with nitrogen gas for 30 s and sealed with a Teflon screw cap. After vigorous vortexing, the samples were placed in the microwave reaction vessel (12 cm × 3 cm I.D., 0.5 cm thickness; Milestone Stard D, Sorisole Bergamo, Italy) containing 30 ml Milli-Q water. The vessels were heated from 25 to 60 °C (800 W for 24 vessels) within 6 min, and maintained at this temperature for 10 min. The samples were then cooled to room temperature, and 1.7 ml NaCl (0.73% w/v) was added to the samples. The samples were then vortexed and centrifuged at 3000×*g* for 10 min, and the organic phase (lower phase) was transferred into a clean tube. Finally, the lipid extracts were dried under vacuum and re-suspended in a chloroform–methanol solution (2:1, v/v) to a final volume of 200 μl, ready for total lipid analysis. The measurement conditions used for the analysis of phospholipids, ergosterol, triacylglycerols and steryl esters with HPLC-CAD were the same as in our previous work [58]. For lipid nomenclature, see Additional file 1: Table S1.

#### Separation of neutral and polar lipids

The protocol used in this study was adapted from the protocol of Löfgren et al. [59]. The lipids obtained from microwave-assisted extraction were dried under vacuum, and the samples then re-suspended in a heptane–methanol mixture (98:2, v/v) to a final volume of 200 μl. After vortexing for 10 min, 1 volume of methanol–water (with 0.23% NH_3_) was added to the solution. The sample was vortexed for a further 10 min at room temperature, after which the upper phase (the heptane phase) was transferred to a clean tube. The lower phase (the methanol phase) was re-extracted twice with heptane (200 μl), and the heptane phases containing the neutral lipids were pooled together. The methanol phase, containing the polar lipids, was transferred to a clean tube. Finally, the solvents (methanol and heptane) were removed by vacuum evaporation, and the dried extracts remaining were used for total FA analysis with GC–MS.

### Analysis of total FAs by GC–MS

The total FAs from the neutral and polar fractions were converted into fatty acid methyl esters (FAMEs), and analyzed using GC–MS, as described in our previous work [60]. Briefly, the dried fractions of neutral and polar lipids were mixed with 800 μl hexane, 400 μl 14% BF_3_ (in methanol) and 20 μg of an internal standard (C17:0) in an extraction tube. The FAs were derivatized to FAMEs using a microwave-assisted method, as described previously [38]. The upper phase (hexane phase) containing FAMEs was analyzed using GC–MS (Focus GC ISQ single quadrupole, Thermo Fisher scientific, Austin, TX). Unknown FAMEs were identified by comparing their retention times and mass spectrum profiles with authentic standards. The unsaturation index was calculated as the sum of the percentage of each unsaturated FA (w/w) multiplied by its number of unsaturated bonds in the mixture [61].

#### Plasmid construction

*OLE1* encoding ∆ (9) FA desaturase (GenBank Accession Number: NC_001139.9) was amplified from genomic DNA of *S. cerevisiae* CEN.PK 113-7D using high-fidelity DNA polymerase (Thermo Fisher Scientific) with the primers OLEF (5′-CGC*GGATCC*ATGCCAACTTCTGGAACTACTAT-3′) and OLER (5′-CCC*AAGCTT* TTAAAAGAACTTACCAGTTTCGTA-3′). A 1533-bp PCR fragment including the entire coding region was obtained and then inserted into the 2-micron plasmid p426TEF [62] under the TEF promoter with *Bam*HI/*Hind*III to yield the plasmid p426TEF-OLE1.

The expression levels of ∆ (9) FA desaturase and squalene epoxidase (*ERG1*) were knocked down using the antisense oligonucleotide method, as described previously [63]. The antisense oligonucleotide of the conserved catalytic domain of *OLE1* was created by annealing the primer pair ELOF (5′-CCC*AAGCTT*TGGGGCCACTCTCACAGAATTCACC ATCGTTAC-3′)/ELOR (5′-CGC*GGATCC*GTAACGATGGTGAATTCTGTGAGAGTGGCCCCA-3′). The antisense oligonucleotide of the conserved catalytic domain of *ERG1* was created similarly using the annealing primer pair ELOF (5′-CCC*AAGCTT*CGATTGTGTCAACAAACCCGTTGAATTTCTGTC-3′)/ELOR (5′-CGC*GGATCC*GACAGAAATTCAACGGGTTTGTTGACACAATCG-3′). The two 33-bp antisense DNA fragments, thus, obtained were then inserted into the plasmid p426TEF under the TEF promoter with *Bam*HI/*Hind*III, separately, forming the plasmids p426TEF-ROLE1 and p426TEF-RERG1.

### Strain construction

Yeast transformations were performed as described by Gietz and Schiestl [64]. *S. cerevisiae* CEN.PK 113-5D was transformed with p426TEF-OLE1, p426TEF-ROLE1 and p426TEF-RERG1, separately. *S. cerevisiae* CEN.PK 113-5D harboring empty p426TEF was used as the control. Transformants were then selected on yeast nitrogen base plates with the addition of amino acids. Transformants harboring the relevant plasmid were confirmed by plasmid extraction and PCR. The depression of *OLE1* and *ERG1* expression was confirmed using qPCR. The *S. cerevisiae* strains harboring p426TEF-OLE1, p426TEF-ROLE1 or p426TEF-RERG1 were designated CEN-O1 (*P*_*TEF*_-*OLE1*), CEN-RO1 (*P*_*TEF*_-*OLE1*-reverse) and CEN-RE1 (*P*_*TEF*_-*ERG1*-reverse), respectively.

### Acid-tolerance test using high-throughput screening

High-throughput toxicity screening was performed using Bioscreen C MBR (Oy Growth Curves AB Ltd, Helsinki, Finland) to determine the appropriate range of each acid to better illustrate the tolerance of the engineered yeast. Recombinant strains harboring p426TEF-OLE1, p426TEF-ROLE1 or p426TEF-RERG1 and the control were pre-cultured in defined medium (as described above) until the exponential growth phase, and then transferred into a 100-well plate containing 120 μl defined medium per well at pH 5.0, with the addition of each acid. The initial OD was about 0.1. Different concentrations of formic (25–250 mM), acetic (25–250 mM), levulinic (50–400 mM) and cinnamic (0.2–1 mM) acid were tested. The 100-well plate was incubated at 30 °C with continuous shaking. The duration of the lag phase and the maximum specific growth rate (*μ*_max_) are presented as mean values of at least five biological replicates ± SD.

### Viability of the yeast cells under acid stress

The yeast transformants in which *OLE1* was overexpressed or *OLE1* or *ERG1* was knocked down, as well as the control, were cultivated until the exponential growth phase. Yeast cells were then recovered and washed twice with sterile water, and re-suspended in defined medium without glucose. These cell suspensions were transferred into defined medium without glucose containing 150 mM formic acid, 150 mM acetic acid, 200 mM levulinic acid or 0.8 mM cinnamic acid at pH 5.0, to yield an OD of 1.0.

To investigate the effect of cellular ergosterol content on acid tolerance, *S. cerevisiae* CEN.PK 113-7D (wild type) was grown on defined medium until the exponential growth phase. The cells were recovered and washed twice with sterile water, and re-suspended in defined medium without glucose. These cell suspensions were transferred into defined medium containing 5 g/l glucose and 10.0 μg/ml ergosterol [44] with the addition of 150 mM formic acid, 150 mM acetic acid, 200 mM levulinic acid or 0.8 mM cinnamic acid at pH 5.0, under oxygen-limited conditions, to yield an OD of 1.0. Yeast cells cultivated under the same stress conditions but without the addition of ergosterol were used as controls. Samples were taken from acid-stressed cultures at various times over a 24-h period. Cell viability was determined by colony counts on YPD plates. Colonies were counted after 2 days’ incubation at 30 °C, and the viability of the cells is reported as the percentage of surviving yeast cells over time.

## Additional file

**Additional file 1: Fig S1.** Comparison of the ethanol and biomass production, and glucose consumption of the yeast strain during aerobic culture without acid (a), and with the addition of 180 mM acetic acid (b), 180 mM formic acid (c), 260 mM levulinic acid (d) and 0.7 mM cinnamic acid (e), at pH 5.0. The first dashed line on the left shows the time at which the acid was pulsed into the culture. Typically, growth phases are defined as: phase 0 (P0), the exponential growth phase before acid addition; phase 1 (P1), the adaptation phase on glucose after acid addition; phase 2 (P2), the exponential growth phase on glucose; phase 3 (P3), the adaptation phase on ethanol; phase 4 (P4), the exponential growth phase on ethanol; and phase 5 (P5), the stationary phase, as has been indicated (b). **Fig S2.** Viable fractions of the *S. cerevisiae* control strain and recombinant strains CEN-RO1 (*P*_*TEF*_-*OLE1*-reverse) and CEN-O1 (*P*_*TEF*_-*OLE1*), under stress resulting from (a) 150 mM acetic acid, (b) 150 mM formic acid, (c) 200 mM levulinic acid and (d) 0.8 mM cinnamic acid, at pH 5.0. **Fig S3.** Expression levels of the key genes in lipid metabolism of *S. cerevisiae* CEN.PK 113-7D in aerobic cultures before (CT, control condition, exponential growth phase) and after the addition of 180 mM acetic acid (AC), 180 mM formic acid (FA), 260 mM levulinic acid (LA) and 0.7 mM cinnamic acid (CA), at pH5.0 (samples were taken 1 h after the addition of the acid). The qPCR results were normalized to *TAF10* and compared with the expression level of each target gene under non-stressed condition. **Fig S4.** Intracellular oxidation level of *S. cerevisiae* CEN.PK 113-7D in aerobic cultures without acid and with the addition of 180 mM acetic acid, 180 mM formic acid, 260 mM levulinic acid and 0.7 mM cinnamic acid, at pH5.0. (a) Adaptation phase on glucose, (b) glucose growth phase, (c) adaptation phase on ethanol, (d) ethanol growth phase and (e) stationary phase. **Table S1.** Lipid classes in *S. cerevisiae*
