# Supplementary material for: Changes in lipid metabolism convey acid tolerance in Saccharomyces cerevisiae
Source: Biotechnol Biofuels. 2018 Oct 29;11:297. doi: 10.1186/s13068-018-1295-5 (PMC6206931; doi:10.1186/s13068-018-1295-5)
Supplement: Supplementary file 1 — Additional file 1: Fig S1. Comparison of the ethanol and biomass production, and glucose consumption of the yeast strain during aerobic culture without acid (a), and with the addition of 180 mM acetic acid (b), 180 mM formic acid (c), 260 mM levulinic acid (d) and 0.7 mM cinnamic acid (e), at pH 5.0. The first dashed line on the left shows the time at which the acid was pulsed into the culture. Typically, growth phases are defined as: phase 0 (P0), the exponential growth phase before acid addition; phase 1 (P1), the adaptation phase on glucose after acid addition; phase 2 (P2), the exponential growth phase on glucose; phase 3 (P3), the adaptation phase on ethanol; phase 4 (P4), the exponential growth phase on ethanol; and phase 5 (P5), the stationary phase, as has been indicated (b). Fig S2. Viable fractions of the S. cerevisiae control strain and recombinant strains CEN-RO1 (PTEF-OLE1-reverse) and CEN-O1 (PTEF-OLE1), under stress resulting from (a) 150 mM acetic acid, (b) 150 mM formic acid, (c) 200 mM levulinic acid and (d) 0.8 mM cinnamic acid, at pH 5.0. Fig S3. Expression levels of the key genes in lipid metabolism of S. cerevisiae CEN.PK 113-7D in aerobic cultures before (CT, control condition, exponential growth phase) and after the addition of 180 mM acetic acid (AC), 180 mM formic acid (FA), 260 mM levulinic acid (LA) and 0.7 mM cinnamic acid (CA), at pH5.0 (samples were taken 1 h after the addition of the acid). The qPCR results were normalized to TAF10 and compared with the expression level of each target gene under non-stressed condition. Fig S4. Intracellular oxidation level of S. cerevisiae CEN.PK 113-7D in aerobic cultures without acid and with the addition of 180 mM acetic acid, 180 mM formic acid, 260 mM levulinic acid and 0.7 mM cinnamic acid, at pH5.0. (a) Adaptation phase on glucose, (b) glucose growth phase, (c) adaptation phase on ethanol, (d) ethanol growth phase and (e) stationary phase. Table S1. Lipid classes in S. cerevisiae [file 13068_2018_1295_MOESM1_ESM.docx]

**Additional file 1**


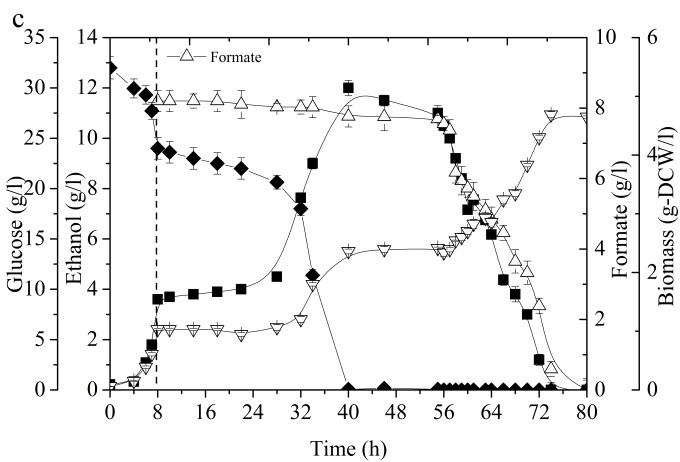

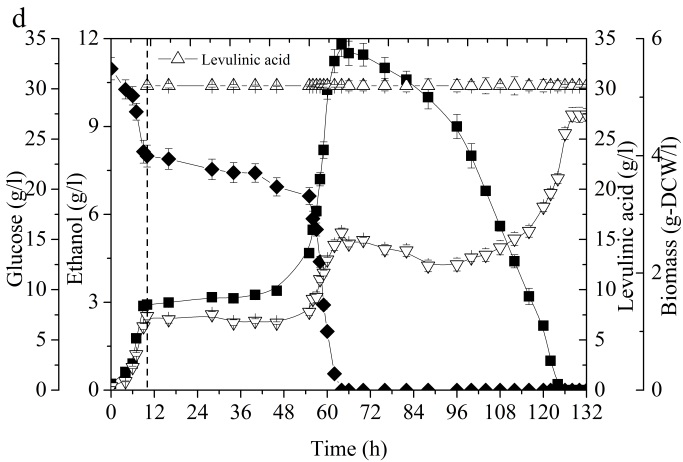

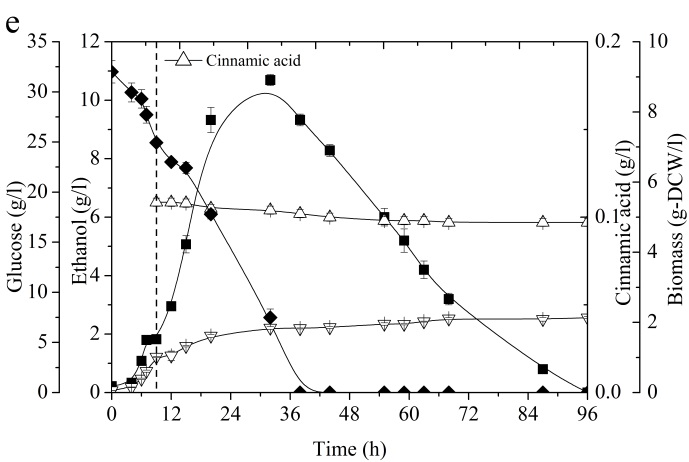

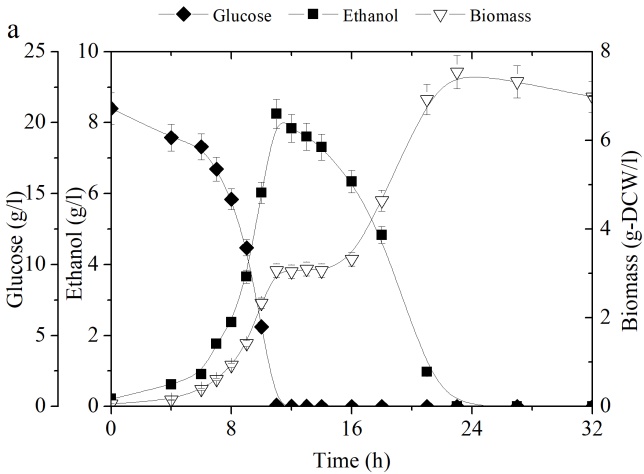

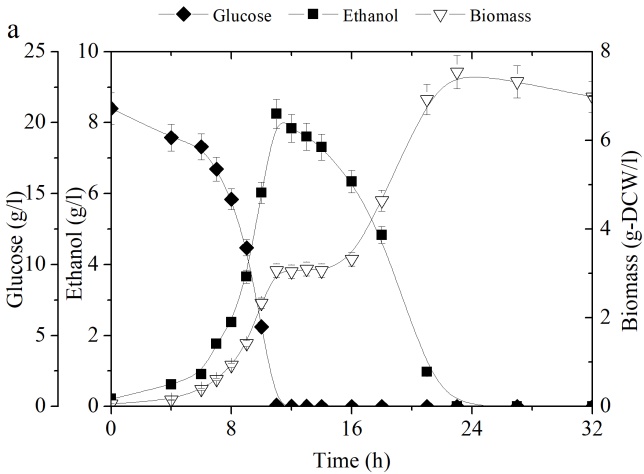

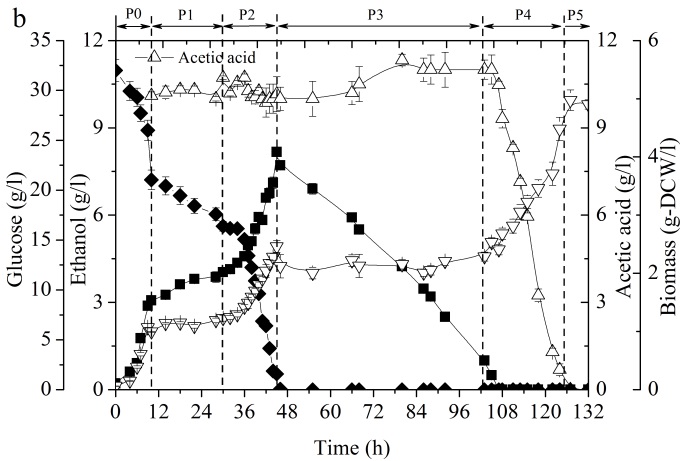


Fig. S1. Comparison of the ethanol and biomass production, and glucose consumption of the yeast strain during aerobic culture without acid (a), and with the addition of 180 mM acetic acid (b), 180 mM formic acid (c), 260 mM levulinic acid (d) and 0.7 mM cinnamic acid (e), at pH 5.0. The first dashed line on the left shows the time at which the acid was pulsed into the culture. Typically, growth phases are defined as: Phase 0 (P0), the exponential growth phase before acid addition; Phase 1 (P1), the adaptation phase on glucose after acid addition; Phase 2 (P2), the exponential growth phase on glucose; Phase 3 (P3), the adaptation phase on ethanol; Phase 4 (P4), the exponential growth phase on ethanol; and Phase 5 (P5), the stationary phase, as has been indicated (b).


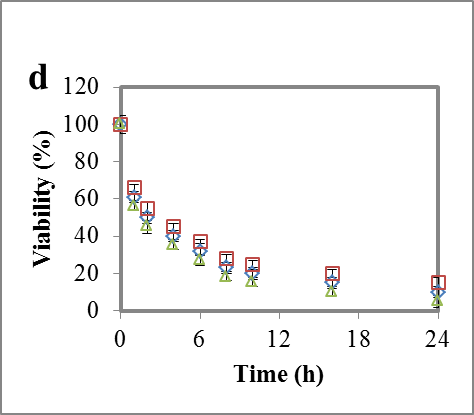

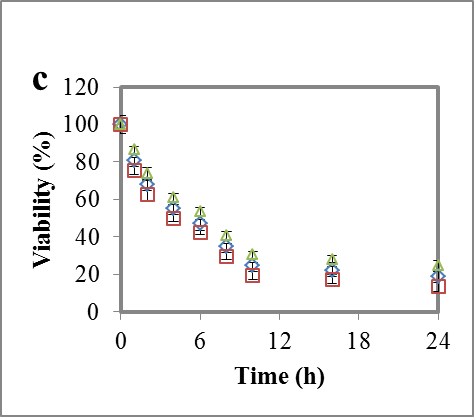

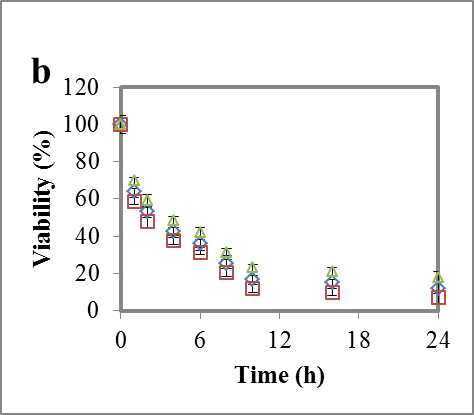

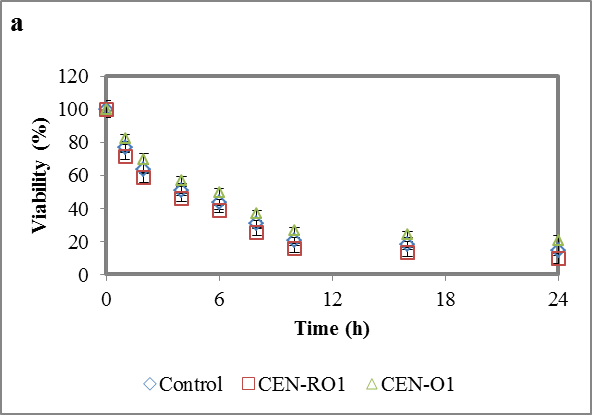

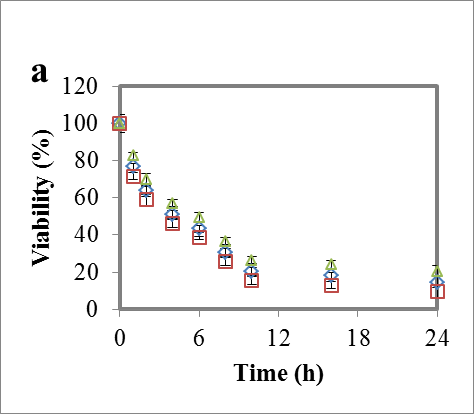


Fig. S2. Viable fractions of the *S. cerevisiae* control strain and recombinant strains CEN-RO1 (*P_TEF_*-*OLE1*-reverse) and CEN-O1 (*P_TEF_*-*OLE1*), under stress resulting from (a) 150 mM acetic acid, (b) 150 mM formic acid, (c) 200 mM levulinic acid and (d) 0.8 mM cinnamic acid, at pH 5.0.


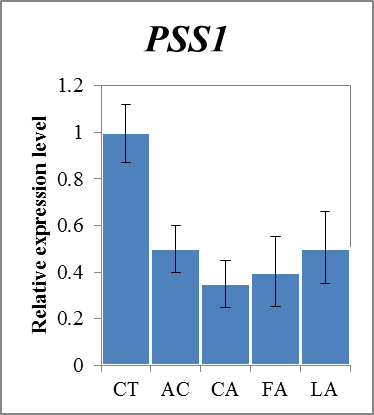

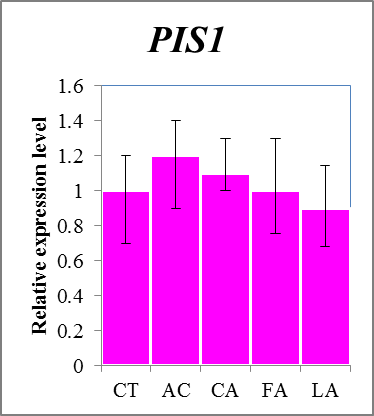

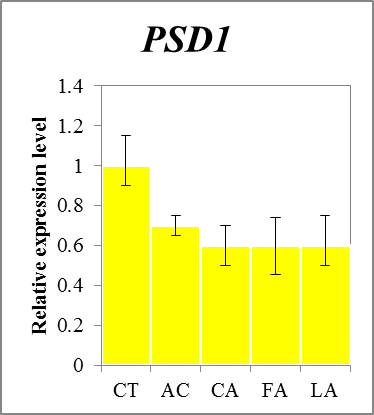

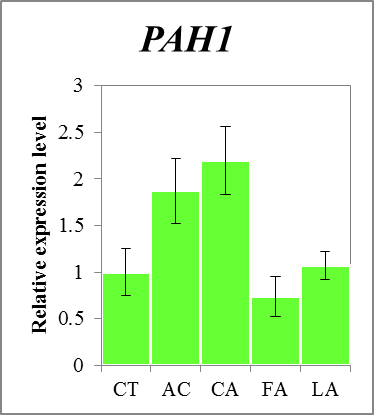

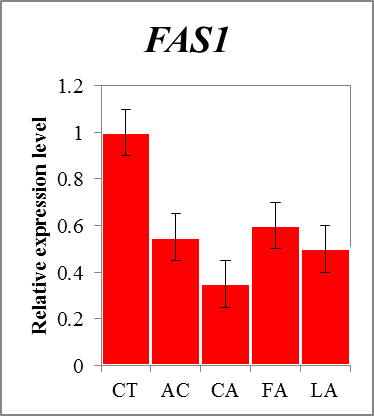

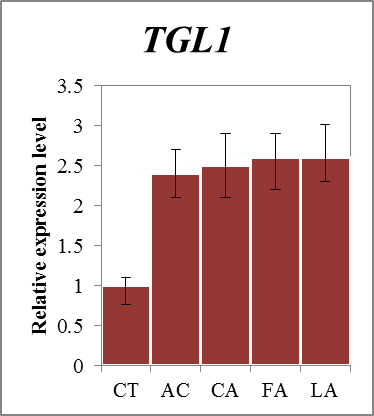

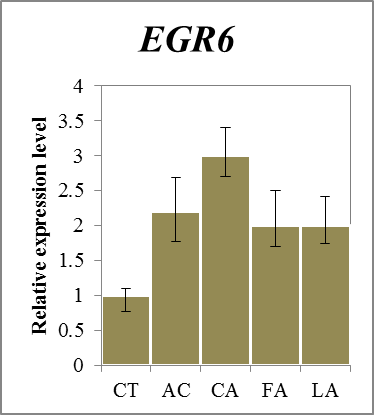

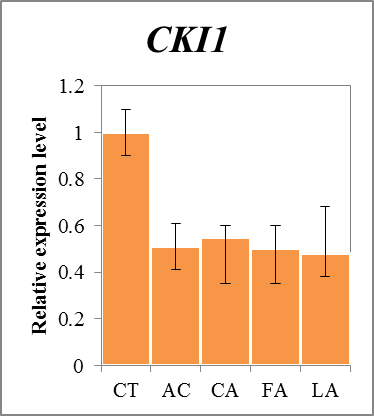

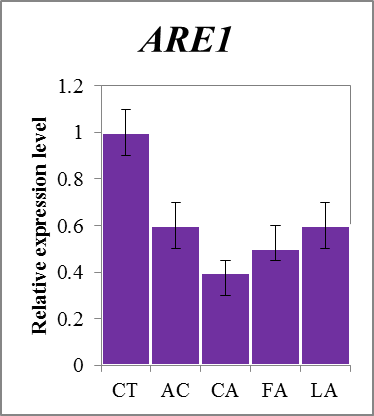

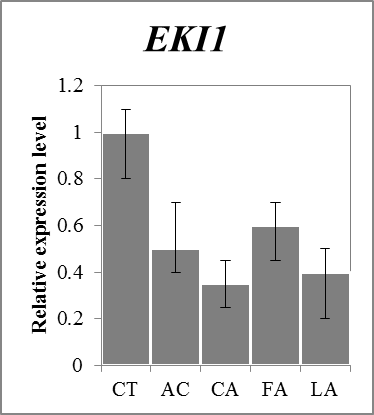

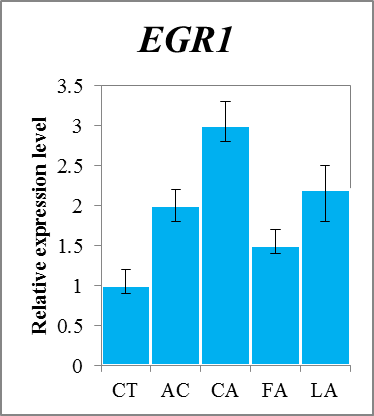
Fig. S3. Expression levels of the key genes in lipid metabolism of *S. cerevisiae* CEN.PK 113-7D in aerobic cultures before (CT, control condition, exponential growth phase) and after the addition of 180 mM acetic acid (AC), 180 mM formic acid (FA), 260 mM levulinic acid (LA) and 0.7 mM cinnamic acid (CA), at pH5.0 (samples were taken 1 h after the addition of the acid). The qPCR results were normalized to *TAF10* and compared with the expression level of each target gene under non-stressed condition.

Fig. S4. Intracellular oxidation level of *S. cerevisiae* CEN.PK 113-7D in aerobic cultures without acid and with the addition of 180 mM acetic acid, 180 mM formic acid, 260 mM levulinic acid and 0.7 mM cinnamic acid, at pH5.0. (a) Adaptation phase on glucose, (b) glucose growth phase, (c) adaptation phase on ethanol, (d) ethanol growth phase and (e) stationary phase.


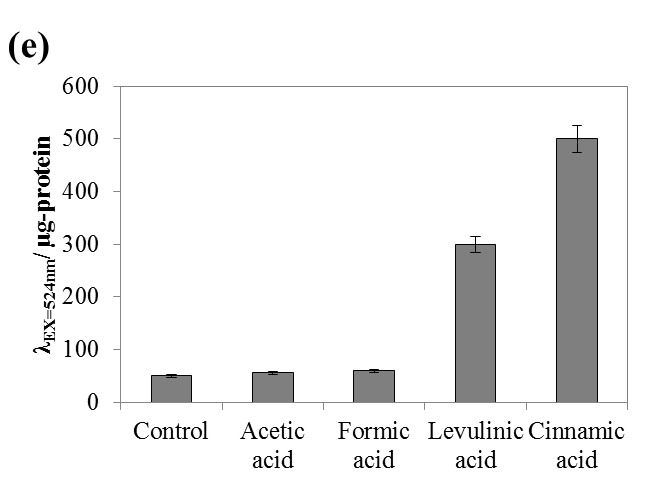

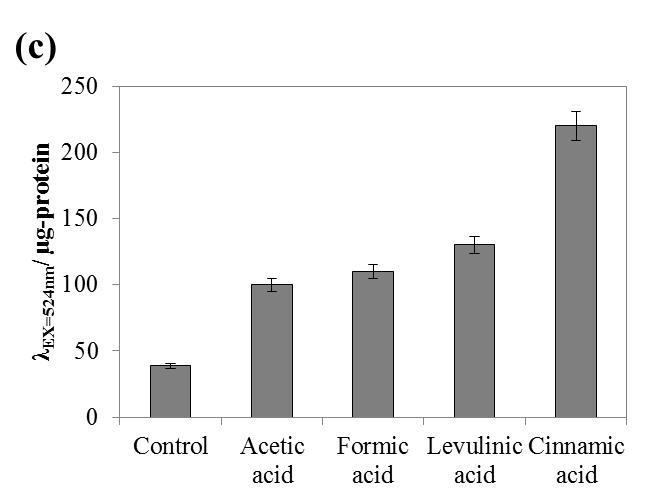

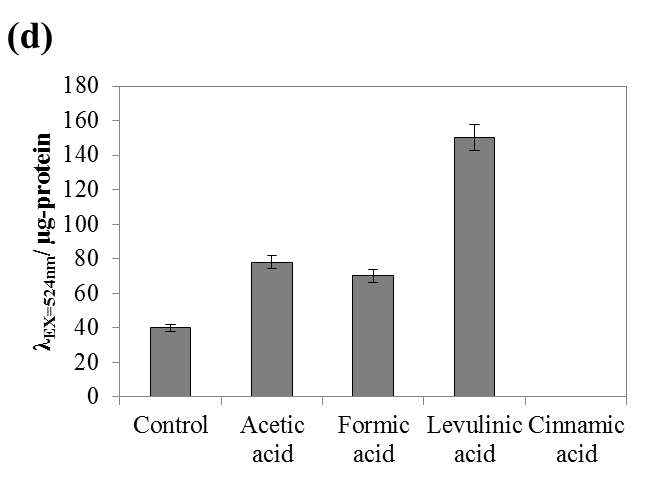

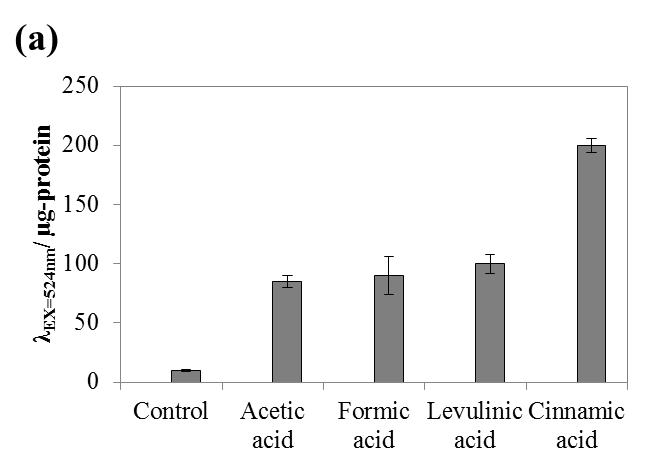

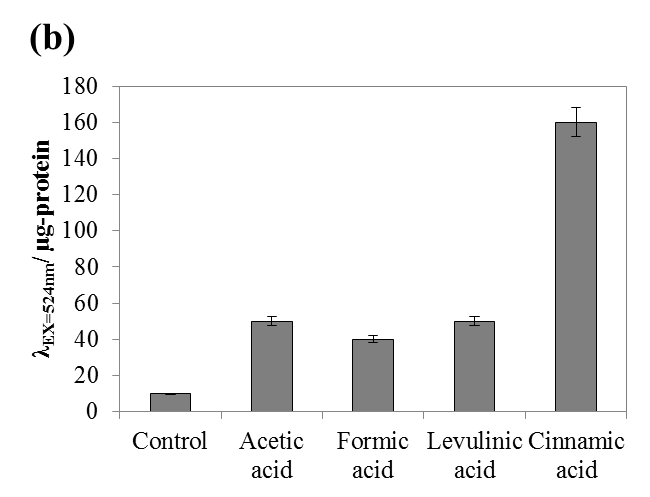


Table S1. Lipid classes in *S. cerevisiae*

| Lipid class | Abbreviation |
| --- | --- |
| Plasma membrane |  |
| phospholipids |  |
| Cardiolipin | CL |
| Phosphatidic acid | PA |
| Phosphatidylcholine | PC |
| Phosphatidylethanolamine | PE |
| Phosphatidylinositol | PI |
| Phosphatidylserine | PS |
| Sterols (Neutral) |  |
| Ergosterol | ES* |
| Lipid droplets (Neutral) |  |
| Triacylglycerols | TAGs |
| Steryl esters | STEs |
